# KZ-BD: Dataset of Kazakhstan banknotes with annotations

**DOI:** 10.1016/j.dib.2024.110076

**Published:** 2024-01-24

**Authors:** Ualikhan Sadyk, Rashid Baimukashev, Akgul Bozshina, Cemil Turan

**Affiliations:** SDU University, Kaskelen, Kazakhstan

**Keywords:** Central Asian currency, Banknote recognition, Currency detection, Machine learning

## Abstract

The field of deep learning is rapidly advancing and impacting various industries, including banking. However, there are still challenges when it comes to accurately identifying the denomination of currencies, especially when dealing with issues like variation within the same class of currency and inconsistent lighting conditions. One notable problem is the lack of available data for Kazakhstan's currency. This research paper introduces the Kazakhstan Banknotes Dataset (KZ-BD), which is a unique collection of 4200 carefully annotated images covering 14 different categories. The dataset includes high-resolution images of authentic Kazakhstan Tenge in both coin and paper note forms, ranging from 1 to 20,000 tenge denominations. Each image has undergone strict de-identification and validation procedures, and the dataset is openly accessible to artificial intelligence researchers. This contribution addresses the data gap in deep learning research related to currency identification by offering a comprehensive dataset for Kazakhstan's currency, enabling better evaluation and fine-tuning of machine learning models with real-world data.

Specifications Table

**Table:** 

Subject	Machine Learning / Deep Learning
Specific subject area	Machine Learning in Currency Recognition
Data format	Raw Annotated
Type of data	Kazakhstan Currency images
Data collection	The data were collected using a high-resolution phone camera to capture images of various denominations of Kazakhstan Tenge banknotes and coins. Rigorous efforts were made to ensure the authenticity of the currency items photographed. Each image was meticulously annotated, highlighting key features for currency recognition. No specific inclusion/exclusion criteria were applied, as the aim was to cover all current denominations of the currency. The data collected remained unaltered without any normalisation process to retain the original quality and details of the images. Subsequent to image capture, a comprehensive de-identification and validation process was conducted to ensure anonymity and data integrity.
Data source location	SDU University Abylaikhan 1/1, Kaskelen, Kazakhstan
Data accessibility	Repository name: Dataset Of Kazakhstan Banknotes With Annotations Data identification number: 10.17632/dny3dgvvw8.1 Direct URL to data: https://data.mendeley.com/datasets/dny3dgvvw8/1
Related research article	U. Sadyk, C. Turan and R. Baimukashev, ``Overview of Deep Learning Models for Banknote Recognition,'' 2023 17th International Conference on Electronics Computer and Computation (ICECCO), Kaskelen, Kazakhstan, 2023, pp. 1-5, doi:10.1109/ICECCO58239.2023.10147142.

## 1 Value of the Data

• The dataset is a comprehensive collection of 4200 high-quality images spanning 14 distinct categories. It is unique in that it includes both coins and paper notes of the Kazakhstan Tenge, making it extremely valuable for developing applications related to currency classification and detection, particularly for Kazakhstan's currency.
• This dataset is indispensable for researchers in the field of currency identification, promoting a more inclusive global AI community.
• The KZ-BD can cater to a wide range of users, including visually impaired individuals who require assistance with identifying currency, banking institutions looking for automated solutions, and government agencies responsible for overseeing financial systems.
• It serves as a robust foundation for training, testing, and validating machine learning models designed for currency classification and identification.
• By filling the existing data gap, the KZ-BD expedites research in currency recognition, encouraging the evaluation and fine-tuning of models using real-world data. Moreover, this dataset is a valuable resource for educators in AI and computer science disciplines, offering well-structured real-world data for teaching and learning purposes.
• The data set is balanced composition across various denominations, diverse environmental conditions, and a variety of image types. The KZ-BD is not only instrumental in currency recognition but also serves as an ideal benchmark for evaluating different neural network architectures that focus on image recognition, detection, processing, and other tasks that involve banknotes and currencies.

## 2 Background

This dataset was compiled to encompass a comprehensive range of banknote images from Kazakhstan, representing various denominations. Its goal is to make it easier to create and assess computer vision algorithms that are specifically designed to recognize and categorise banknotes according to their denomination.This dataset was created because it was needed to support strong machine learning models that could recognize and categorise banknotes with accuracy. These models are essential for use in cash counting devices, automated teller machines (ATMs), and financial technology improvements.

In order to guarantee the dependability of the model, we concentrated on selecting a wide range of images, including banknotes taken from different perspectives, in different lighting, and with different orientations.

### 2.1 Data description

The KZ-BD dataset has several important functions. It plays a crucial role in accurately identifying currency denominations, which is essential for automated financial systems and counterfeit detection. Additionally, it aids visually impaired individuals who struggle to recognise currency denominations [1].

This dataset comprises 4200 high-quality colour images, organised into 14 distinct categories based on the denominations of the Kazakhstan Tenge, ranging from 1 to 20,000 Tenge [2].

Of the 14 classes within the dataset, 8 classes represent various coin denominations, while the remaining 6 classes depict paper notes.The images were captured under diverse lighting conditions and against various backgrounds to closely mimic real-world usage. These backgrounds include dark, illuminated, cluttered, occluded and folded settings. Moreover, two types of images were taken for each banknote – one from the front and one from the back. These images are available in the widely compatible .jpg file format, suitable for the majority of researchers.

The KZ-BD dataset is conveniently packaged in a single zip file named ``Kazakhstan_Banknotes_Dataset.zip''. Upon extraction, you'll find a main folder named ‘Kazakhstan_Banknotes_Dataset,’ containing two essential subfolders: ‘training’ and ‘validation.’ The ‘training’ folder is meant for training machine learning models, while the ‘validation’ folder is designed for testing these models.

For both training and validation purposes, there are a total of 14 subfolders, each representing a specific banknote denomination. This structured hierarchy makes it effortless for researchers to navigate and locate the specific images they need for their research.

**Fig. 1:**
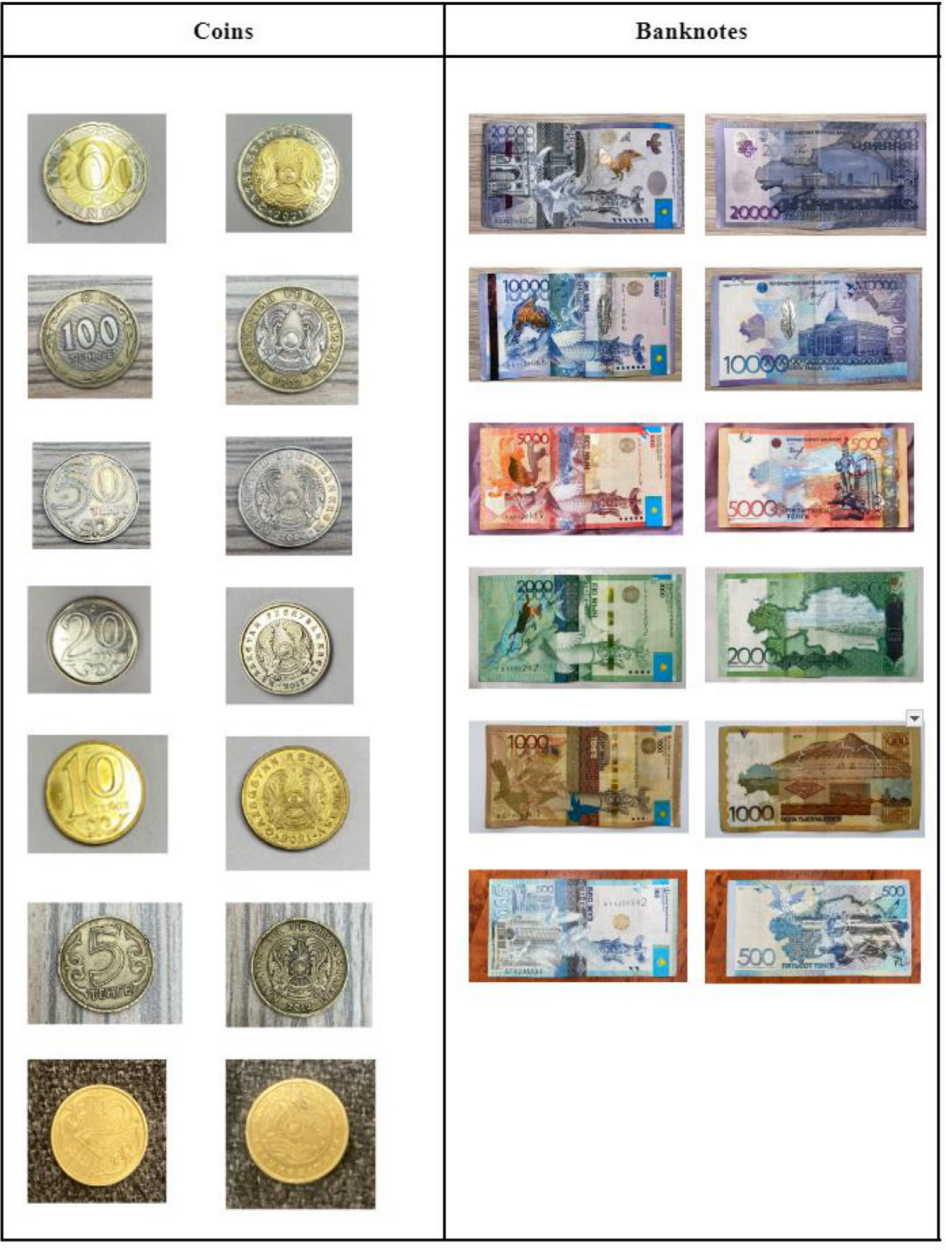
Visual Samples of "Kazakhstan_Banknotes_Dataset".

Fig. 1 displays a collection of data samples from the ``Kazakhstan_Banknotes_Dataset,'' showcasing images representing various currency denominations. The Fig. features both coins on the left and banknotes (paper notes) on the right, offering a clear and detailed portrayal of Kazakhstan's banknotes and coins.

**Fig. 1.1:**
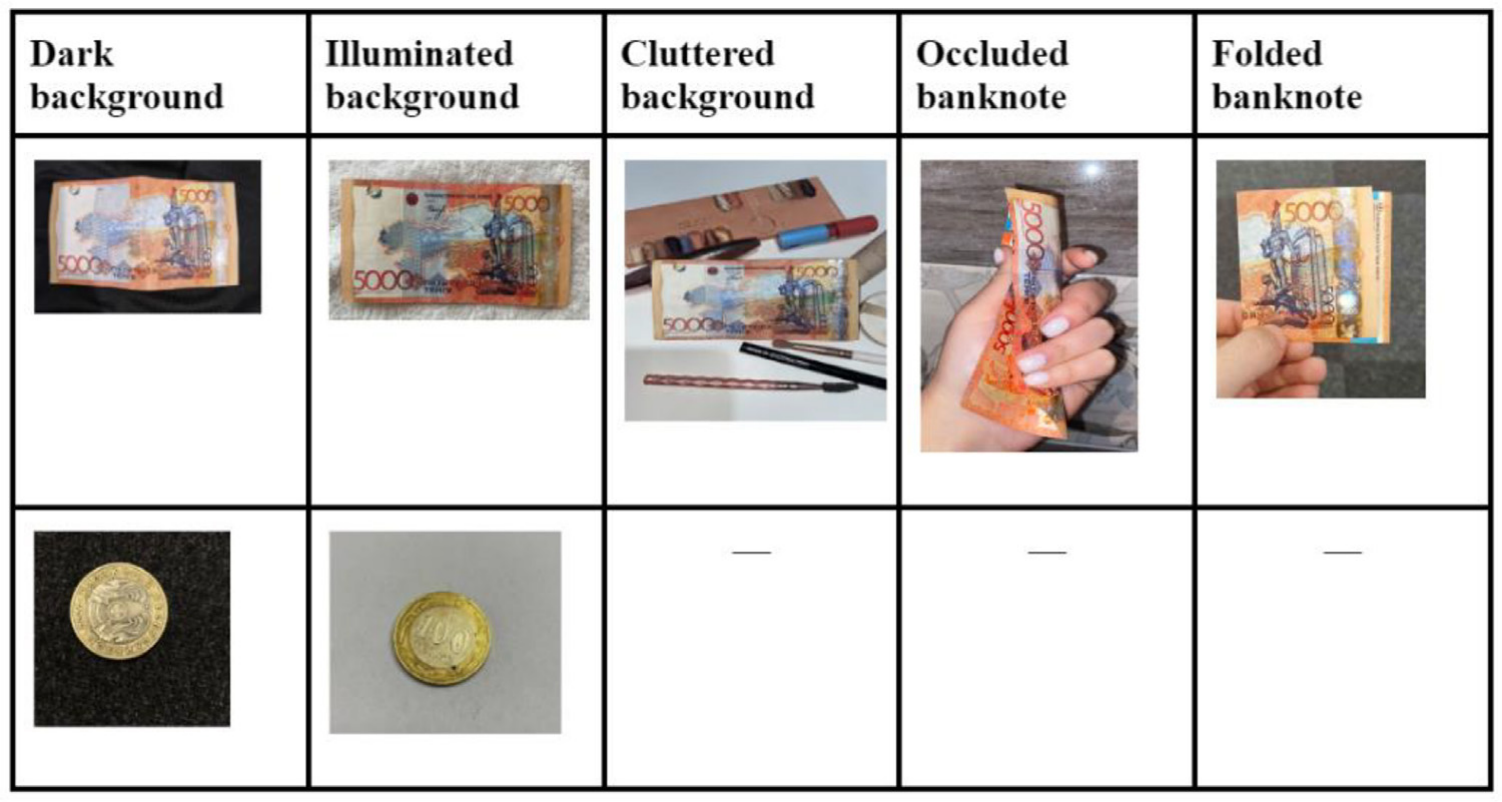
Banknote images taken in various environments.

Fig. 1.1 displays sample images from the dataset, showcasing images captured in various environmental conditions, providing a holistic representation of the dataset's robustness to different scenarios. These conditions include dark backgrounds, illuminated backgrounds, cluttered backgrounds, occluded banknotes, and folded banknotes.

For the paper banknotes category, Fig. 1.1 demonstrates their appearance against dark backgrounds, illuminated backgrounds, cluttered backgrounds, occluded and folded conditions. Similarly, for the coins category, the figure showcases their presentation against dark backgrounds and illuminated backgrounds.

Figs. 1 and 1.1 together offer a comprehensive and detailed overview of the ``Kazakhstan_Banknotes_Dataset,'' encompassing both the currency categories and the environmental conditions in which the dataset was captured.

### 2.2 Image annotation

In machine learning, image annotation is essential because it helps algorithms understand and classify images by accurately recognizing objects within the images. In order to properly classify and define objects of interest for machine learning models, bounding boxes are placed strategically around them in images.

A common and simple to use annotation tool named LabelImg was used to annotate the Kazakhstan banknotes dataset. Using LabelImg, annotators can manually label objects of interest within images to generate bounding boxes around them. In order to determine the object's boundary, this method involves selecting specific regions and simultaneously marking the object's class and spatial dimensions.

The banknote images and their annotations in text format for Kazakhstan are stored in the “Kazakhstan_Banknotes_Dataset” folder, serving as the main directory.

Each line in the annotation file is a unique bounding box that defines various elements of the banknote images. The YOLO format is used to save these annotations in text files with the following format [[3], [4]]:

<class> <x-min>, < y-min>, <width >, <height>

1. **<class>**: Represents the class of the annotated object.
2. **<x-min>**: Denotes the normalised x-coordinate of the top-left corner of the bounding box. It specifies the horizontal position of the box's left edge relative to the image's width.
3. **<y-min>**: Represents the normalised y-coordinate of the top-left corner of the bounding box. It specifies the vertical position of the box's top edge relative to the image's height.
4. **<width>**: Indicates the normalised width of the bounding box, representing how wide the bounding box is relative to the image's width.
5. **<height>**: Represents the normalised height of the bounding box, indicating how tall the bounding box is relative to the image's height.

**Fig. 1.2:**
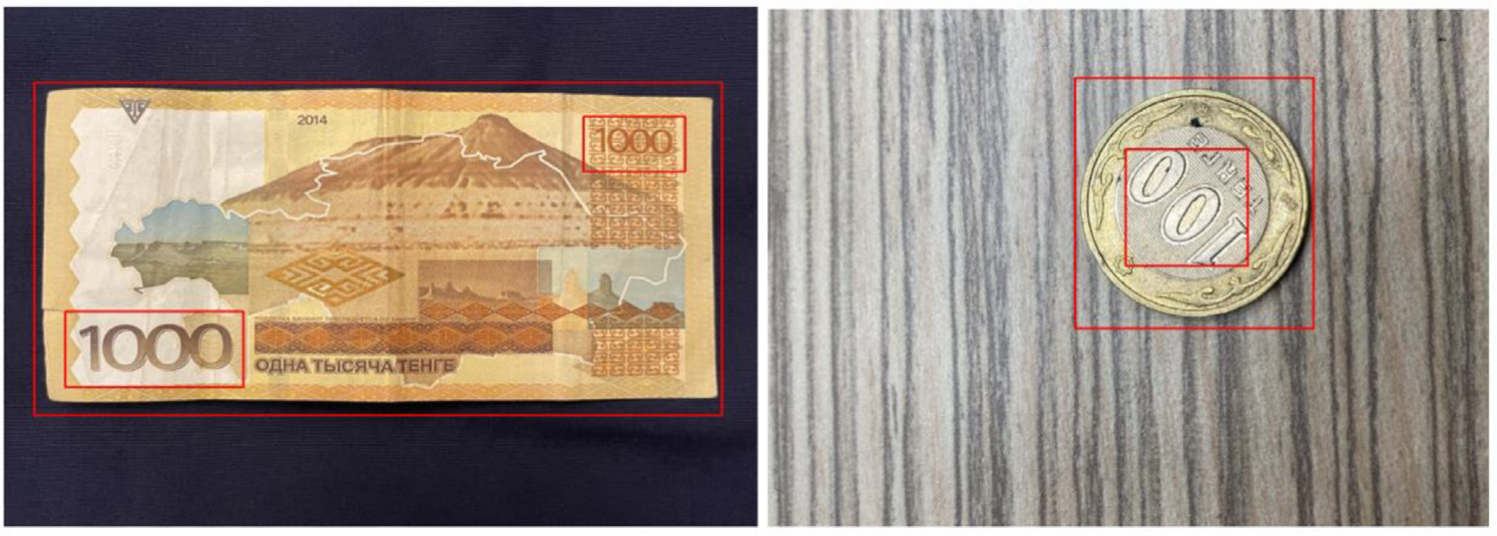
Banknote images with bounding box annotations.

Fig. 1.2 represents a sample screenshot of the banknote images with corresponding bounding box annotations.

**Outer Bounding Box**: Represents information for the primary object, such as the 1000 tenge banknote and 100 tenge coin. This outer bounding box encapsulates the primary features of the banknote or coin.

**Bounding Boxes Placed Inside Outer Bounding Boxes**: Correspond to additional bounding boxes for other objects within the image. These inner bounding boxes could signify various elements such as denomination text on the banknote, security features, or any other relevant details. Each line in the annotation file corresponds to a specific bounding box, providing a comprehensive representation of the objects present in the image. It enables the model to learn and recognize various components, contributing to a more comprehensive understanding of the image content.

**Fig. 1.3:**
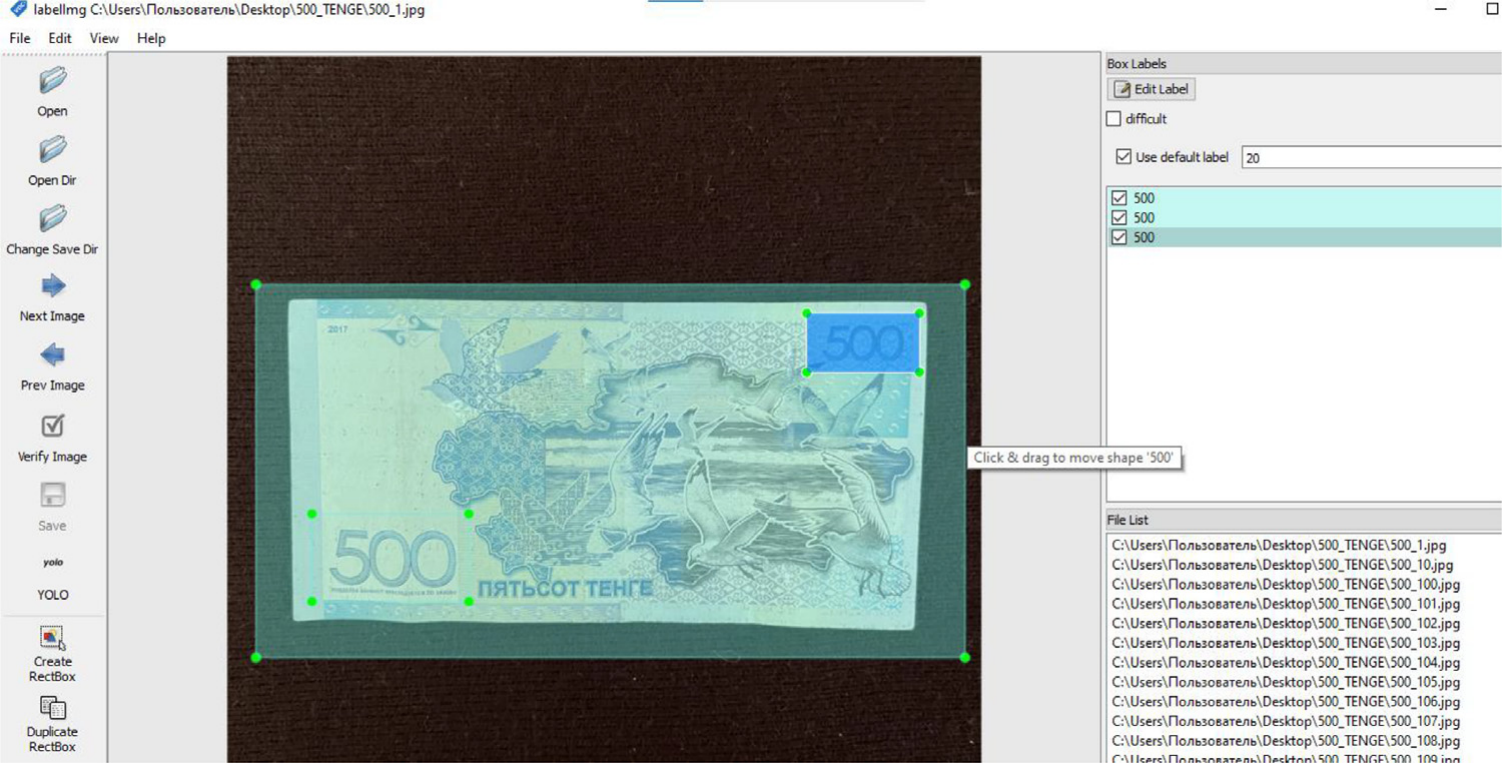
Screenshot of labelimg tool for image annotation.

Furthermore, to provide an overview of the annotation procedure, Fig. 1.3 presents an example screenshot of the banknote images along with the associated bounding box annotations. The annotation process is made easier by the LabelImg tool, which makes it possible for annotators to precisely identify and classify items in images. This helps to create well annotated datasets that are useful for machine learning tasks.

**YOLO Testing Results:**

Extensive testing was conducted on the YOLO formatted annotations to verify their accuracy in banknote recognition and classification. The Kazakhstan banknotes dataset was used to evaluate the model's performance using YOLO (You Only Look Once). Based on the annotated images, the model successfully recognized and classified different currency denominations through testing.

**Fig. 1.4:**
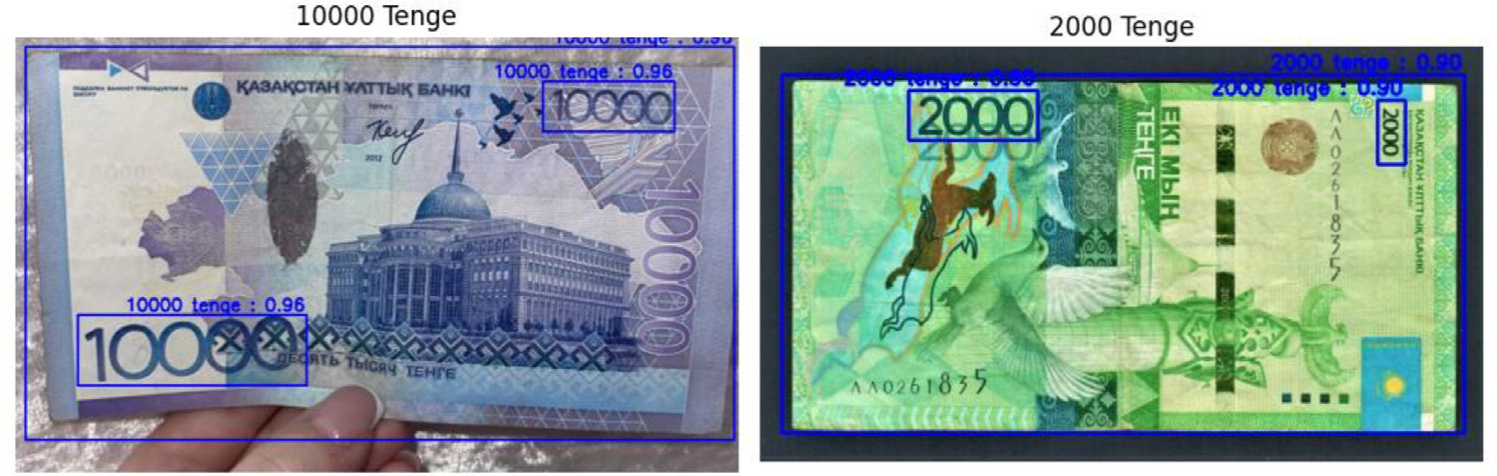
YOLO testing result.

The Fig. 1.4 displays a test scenario where a 2000 and 10000 tenge banknotes are correctly identified and classified by the YOLO model. The bounding box annotations precisely outline the detected banknote, showcasing the model's ability to recognize and categorise the specific denomination within the image. The YOLO model achieved an accuracy rate, correctly identifying the 10000 tenge banknote with an accuracy of 96% and accurately categorising the 2000 tenge banknote with an accuracy rate of 90%. These high accuracy percentages reflect the YOLO model's robustness and reliability in discerning between different currency denominations, reaffirming its effectiveness in accurate banknote classification.

### 2.3 Dataset structure

**Fig. 2:**
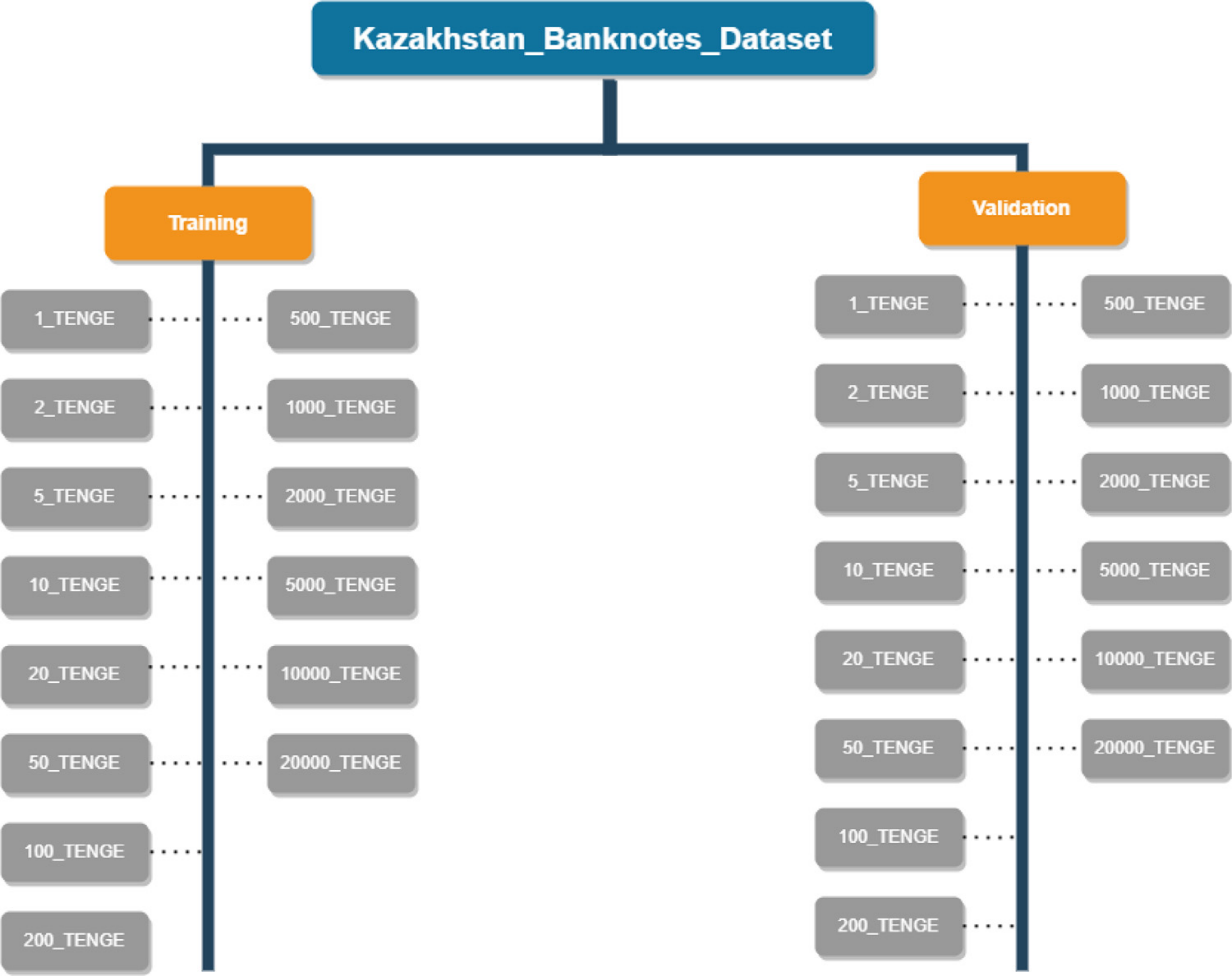
Banknote dataset directory structure.

In Fig. 2, the directory structure of the banknote dataset is depicted. The dataset is organised into two main folders: ``Training'' and ``Validation.''

Within the ``Training'' folder, there are 14 subfolders, each representing a specific denomination of Kazakhstan Tenge banknotes. Similarly, the ``Validation'' folder also contains 14 subfolders, each corresponding to a distinct banknote denomination.

This hierarchical structure ensures that the dataset is neatly categorised, with each subfolder containing the respective images for a particular denomination. Researchers and users can easily navigate through these folders to access the specific images they require for their work, making the dataset highly accessible and user-friendly for tasks such as currency recognition and classification.

To delve deeper into this dataset, let's examine the composition of images within each folder.

### 2.4 The ``training'' folder

Within the ``training'' folder, there are a total of 2,940 images. Remarkably, each denomination subfolder contains precisely 210 images. This balance creates a uniform distribution of denominations for training purposes. In other words, there are 14 distinct classes, each corresponding to a different banknote denomination.

### 2.5 The ``validation'' folder

The ``validation'' folder, on the other hand, contains 1,260 images, evenly distributed among the 14 denominations. Within each denomination subfolder, you will find 90 images.

**Fig. 2.1:**
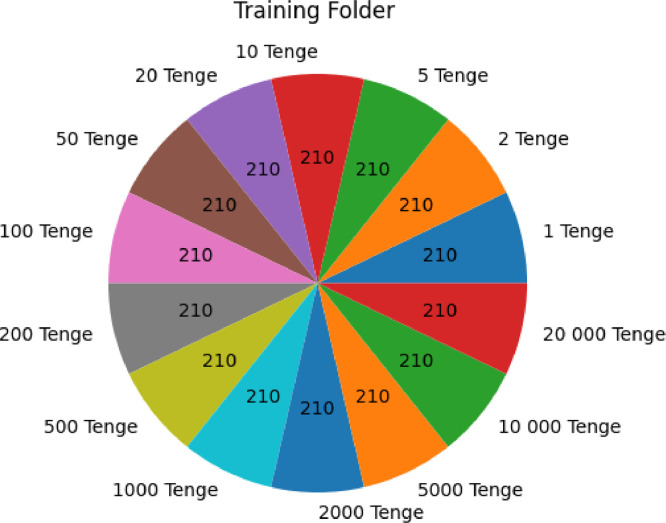
Number of each currency denomination in the training folder of KZ BD dataset.

**Fig. 2.2:**
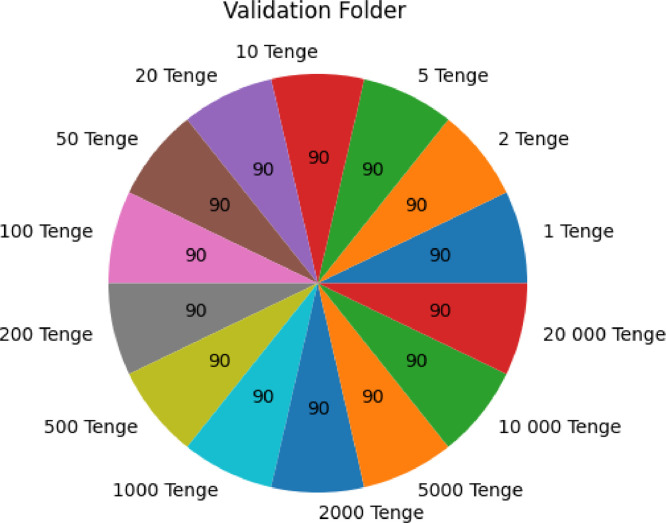
Number of each currency denomination in the validation folder of KZ BD dataset.

Figs. 2.1 and 2.2 provides a visual representation of the dataset's distribution, illustrating the number of images for each denomination presented in both the ``training'' and ``validation'' folders. This analysis highlights how the dataset is structured, with an equal representation of each denomination in both training and validation sets.

## 3 Experimental Design, Materials and Methods

### 3.1 Experimental design

**Fig. 3:**
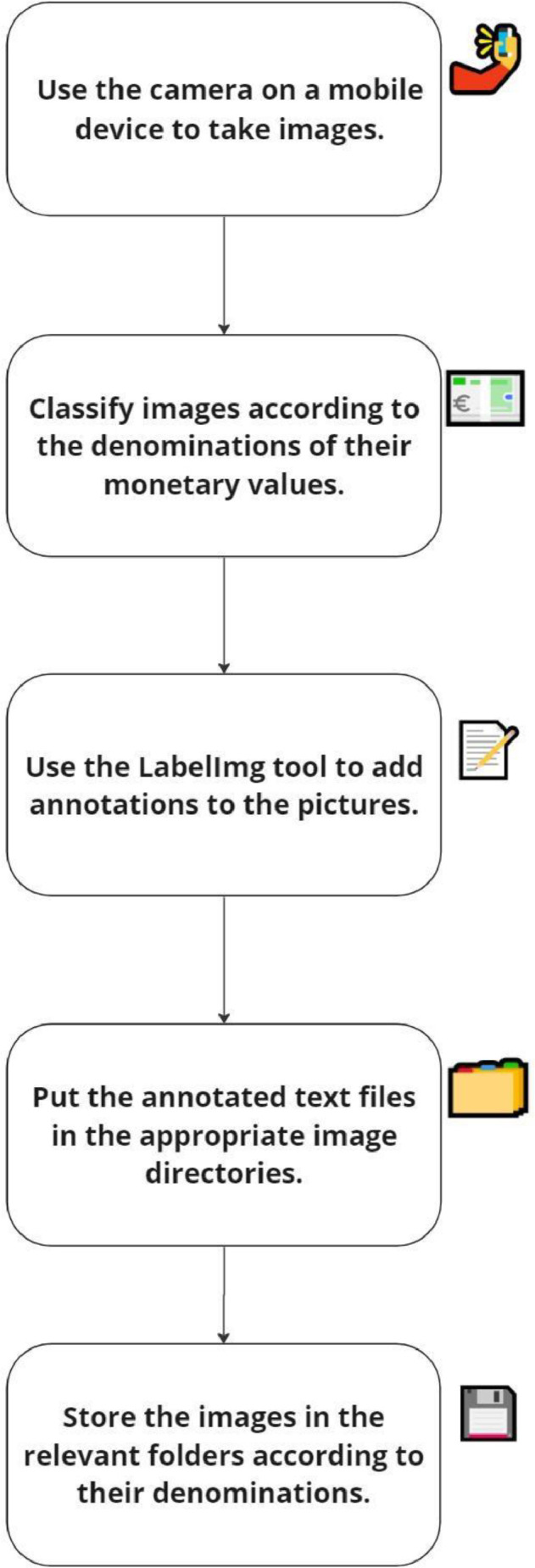
Banknote data acquisition process.

The image data acquisition process, as illustrated in Fig. 3, involved capturing banknote images using the high-resolution rear camera of an iPhone 11 mobile device. A total of 4200 images were taken, all related to Kazakhstan Tenge denominations, with 2400 images representing coins and 1800 images representing paper banknotes. These images were carefully organised and saved into folders based on their respective denomination values.

Table 1 outlines the steps involved in the data acquisition process, while Table 2 provides specific details regarding the image acquisition process. The banknote images were captured during daylight hours in the months of August to September, utilising the iPhone 11 rear camera. The images were taken from various angles and against different backgrounds, as indicated in Fig. 1.1.

**Table 1:** Data acquisition steps.

Step Number	Step Description	Duration	Activity
1.	Collecting Data	August-September	On a daily basis during the day, images of the banknotes were taken.
2.	Annotating Images	September-October	The 4200 images of Kazakhstan banknotes have been annotated

**Table 2:** Specification of image acquisition.

Sp. Number	Specification	Details
1	Camera	(i) Make and Model: Apple iPhone 11 (ii) Rear Camera: 12-megapixel (f/1.8) (iii) Rear autofocus
2	Battery	3110 mAh
3	Annotation Software	LabelImg
4	Image Resolution	1024 × 1024 for images of paper notes 3024 × 4032 for images of coins
5	Image Format	JPG

Once the images of Kazakhstan banknotes were captured, they were sorted into appropriate folders. The detailed folder structure for these images is depicted in Fig. 2. Subsequently, a Python script was employed to resize the banknote images. Finally, the images were annotated using the LabelImg tool from September to October. It's also important to remember that the images were resized throughout the annotating process. The original image sizes were 3024*×*4032 for coins and 1024*×*1024 for paper notes. However, the purpose of downsizing to 1024*×*1024 was to make the annotation procedure with the LabelImg tool easier. The scaling procedure provided conformity with the annotation tool's specifications and played a key role in optimising the annotation process. It's important to point out that the real dataset keeps the original image sizes (3024 *×* 4032 for coins, 1024 *×* 1024 for paper notes), preserving the accuracy of the captured banknotes.

### 3.2 Materials and specifications of image acquisition system

The acquisition of Kazakhstan banknote images was carried out using an Apple iPhone 11 equipped with a 12-megapixel rear camera. The original images of paper notes were captured at 1024 × 1024 pixels, while images of coins were captured at 3024 × 4032 pixels. These images were then saved in .jpg format. The image capture process encompassed a wide range of environmental conditions, including variations in lighting, backgrounds, angles, and scenarios involving folded or partially obscured banknotes.

The Kazakhstan banknotes were further divided into 14 different folders, each dedicated to a specific denomination of Kazakhstan currency. The hierarchical directory structure of the image dataset is illustrated in Fig. 2. The images were subsequently annotated using the LabelImg tool, with both the annotations and the Kazakhstani banknote images being stored within their respective folders.

### 3.3 Methodology

#### Image acquisition

An iPhone 11′s back camera was used to take the currency photos that make up the Kazakhstan Banknotes Dataset (KZ-BD). To guarantee diversity in the dataset, purposeful variation in capturing angels and backgrounds was used. This method aimed to capture nuances and features of banknotes under various lighting and surrounding situations.

#### Annotation process

Using the LabelImg tool, the banknote pictures were carefully annotated. This annotation process was carried out with precision in order to fully label a variety of components inside the banknote images.

### Dataset overview and environmental distribution

Table 3 provides a comprehensive overview of the dataset, including details about the considered denominations, image capture directions (front side or back side of the banknotes/coins), diverse environmental conditions, and the corresponding quantity of images. This thorough tabulation provides an informative overview, showcasing the diversity of the dataset and the variety of environmental situations that were captured.

**Table 3:** Description of the Kazakhstan banknotes dataset (KZ-BD).

	Denomination	Image Capture Direction	Environmental conditions	Number of images	Total images
1	20 000 Tenge (Paper Banknote)	Front side, Back side	Dark Illuminated Cluttered Occluded Folded	86 132 41 21 20	300
2	10 000 Tenge (Paper Banknote)	Front side, Back side	Dark Illuminated Cluttered Occluded Folded	89 142 21 29 19	300
3	5 000 Tenge (Paper Banknote)	Front side, Back side	Dark Illuminated Cluttered Occluded Folded	81 130 32 23 34	300
4	2 000 Tenge (Paper Banknote)	Front side, Back side	Dark Illuminated Cluttered Occluded Folded	82 132 44 20 22	300
5	1 000 Tenge (Paper Banknote)	Front side, Back side	Dark Illuminated Cluttered Occluded Folded	83 128 39 22 28	300
6	500 Tenge (Paper Banknote)	Front side, Back side	Dark Illuminated Cluttered Occluded Folded	79 143 35 13 30	300
7	200 Tenge (Coin)	Front side, Back side	Dark Illuminated	76 224	300
8	100 Tenge (Coin)	Front side, Back side	Dark Illuminated	103 197	300
9	50 Tenge (Coin)	Front side, Back side	Dark Illuminated	105 195	300
10	20 Tenge (Coin)	Front side, Back side	Dark Illuminated	103 197	300
11	10 Tenge (Coin)	Front side, Back side	Dark Illuminated	93 207	300
12	5 Tenge (Coin)	Front side, Back side	Dark Illuminated	170 130	300
13	2 Tenge (Coin)	Front side, Back side	Dark Illuminated	125 175	300
14	1 Tenge (Coin)	Front side, Back side	Dark Illuminated	147 153	300

Additionally, Table 4 displays the distribution of images in the Kazakhstan Banknotes Dataset (KZ-BD) under various environmental conditions. The table shows how many images were taken of coins and banknotes in different conditions, such as dark, illuminated, cluttered, occluded, and folded conditions. This detailed analysis provides an understanding of the diversity of the dataset by showing the number of images taken in different environmental conditions.

**Table 4:** Distribution of images in different environments for coins and banknotes in the Kazakhstan banknotes dataset (KZ-BD).

Environment	Coin Image	Banknotes (paper notes) images	Total Images
Dark	922	500	1422
Illuminated	1478	807	2285
Cluttered	-	212	212
Occluded	-	128	128
Folded	-	153	153
	2400	1800	4200

Total number of Images: 4200

This description will give readers a clear understanding of the structure and composition of the KZ-BD dataset.

## Limitations

Not applicable.

## Ethics Statement

There is no funding allocated for this current project, and there are no conflicts of interest to disclose. The data used is publicly available.

## CRediT authorship contribution statement

**Ualikhan Sadyk:** Conceptualization, Writing – original draft, Writing – review & editing. **Rashid Baimukashev:** Visualization, Investigation. **Akgul Bozshina:** Methodology, Software, Formal analysis. **Cemil Turan:** Supervision, Project administration.

## Data Availability

Dataset Of Kazakhstan Banknotes with Annotations (Original data) (Mendeley Data).
